# Robust oblique Target-rotation for small samples

**DOI:** 10.3389/fpsyg.2023.1285212

**Published:** 2023-11-27

**Authors:** André Beauducel, Norbert Hilger

**Affiliations:** Department of Psychology, University of Bonn, Bonn, Germany

**Keywords:** exploratory factor analysis, factor-rotation, independent clusters model, factor inter-correlation, Target-rotation

## Abstract

**Introduction:**

Oblique Target-rotation in the context of exploratory factor analysis is a relevant method for the investigation of the oblique simple structure. It was argued that minimizing single cross-loadings by means of target rotation may lead to large effects of sampling error on the target rotated factor solutions.

**Method:**

In order to minimize effects of sampling error on results of Target-rotation we propose to compute the mean cross-loadings for each block of salient loadings of the independent clusters model and to perform Target-rotation for the block-wise mean cross-loadings. The resulting transformation-matrix is than applied to the complete unrotated loading matrix in order to produce mean Target-rotated factors.

**Results:**

A simulation study based on correlated independent clusters model and zero-mean cross-loading models revealed that mean oblique Target-rotation resulted in smaller bias of factor inter-correlations than conventional Target-rotation based on single loadings, especially when sample size was small and when the number of factors was large. An empirical example revealed that the similarity of Target-rotated factors computed for small subsamples with Target-rotated factors of the total sample was more pronounced for mean Target-rotation than for conventional Target-rotation.

**Discussion:**

Mean Target-rotation can be recommended in the context of oblique factor models based on simple structure, especially for small samples. An R-script and an SPSS-script for this form of Target-rotation are provided in the Supplementary Material.

## 1 Introduction

Exploratory factor analysis (EFA) is a widely used multivariate method (Harris, 2013), especially in the context of the development of instruments for psychological assessment. Although confirmatory factor analysis may be used for similar purposes, there is still room for EFA because the expectation of perfect simple structure with one large salient loading of each observed variable on one factor and zero cross-loadings, i.e., an independent clusters model (ICM), may lead to unrealistic simplifications in the context of confirmatory factor analysis. The specification of the ICM for data sets with substantial cross-loadings may cause model misfit in confirmatory factor analysis resulting in model modifications and capitalization on chance (MacCallum et al., 1992). Hsu et al. (2014) demonstrated that even small cross-loadings not specified in the confirmatory factor model may result in substantial overestimation of factor covariances. Ximénez et al. (2022) have shown that unspecified cross-loadings in bifactor models may impair parameter recovery and that common fit indices may not be useful for the detection of misfit due to unspecified cross-loadings. Moreover, Hayes and Usami (2020) provide algebraic demonstrations of the bias of factor inter-correlations resulting from unspecified cross-loadings. Overall, these studies indicate that unspecified cross-loadings may cause problems for confirmatory factor analysis and structural equation modeling. Problems with unspecified cross-loadings do not occur with the ICM in the context of EFA because Target-rotation toward an ICM in the context of EFA or exploratory structural equation modeling (ESEM; Asparouhov and Muthén, 2009) will only provide an orientation of the factor axes so that cross-loadings might be minimized without any consequence for model fit. When the cross-loadings are large, Bayesian structural equation modeling (BSEM) may also be superior to confirmatory factor analysis based on the ICM. If the priors are known BSEM might be preferred to ESEM and ESEM might be preferred when the priors are unknown (Wei et al., 2022).

The advantage of using Target-rotation in the context of EFA instead of an ICM in the context of confirmatory factor analysis has been demonstrated for the five-factor model of personality (McCrae et al., 1996). Empirical research has also shown that the use of Target-rotation in the context of ESEM allows to avoid an over-estimation of factor inter-correlations that may occur when the ICM is specified in the context of confirmatory factor analysis (Joshanloo, 2016). The relationship between cross-loadings, factor inter-correlations, and different criteria of factor rotation has also been investigated in the context of simulation studies (Sass and Schmitt, 2010; Schmitt and Sass, 2011). Sass and Schmitt (2010) found that the criteria of factor rotation differ in allowing for larger cross-loadings and in the size of the resulting factor inter-correlations.

The relationship between the loading pattern and the factor inter-correlations has also been addressed by Zhang et al. (2019), who extended partial Target-rotation in order to allow for the specification of a Target-matrix for the factor inter-correlations in addition to the Target-matrix for the loadings. With their extension Target-rotation allows for the investigation of hypotheses on the size of factor inter-correlations. Their approach is based on oblique partial Target-rotation (Browne, 1972) and the gradient projection algorithm (Jennrich, 2002; Bernaards and Jennrich, 2005). Moreover, Hurley and Cattell (1962) initially introduced complete oblique Target-rotation providing rotated loadings and estimates for factor inter-correlations when all values of the Target-matrix of loadings are specified.

While Target-rotation allows for a specification of the ICM in the Target-loadings, Target-rotation will typically be performed in order to minimize cross-loadings. Unless specific Target-values are specified for the correlations by means of extended Target-rotation, Target-rotation will modify the factor inter-correlations in order to reduce cross-loadings. If the ICM holds in the population, sampling error will nevertheless lead to some cross-loadings. When the distribution of cross-loadings resulting from sampling error is not perfectly symmetric, minimizing these cross-loadings may affect the factor inter-correlations. Thereby, sampling error may affect factor inter-correlations resulting from Target-rotation. Moreover, when an ICM holds and when single cross-loadings are minimized by Target-rotation, random differences between single cross-loadings may also affect the rotated loading pattern.

It is therefore proposed to minimize the effect of sampling error on the loading pattern and factor inter-correlations resulting from oblique Target-rotation by means of minimizing mean cross-loadings instead of the single cross-loadings. It is expected that using the mean cross-loadings instead of the single cross-loadings for rotation will reduce the effect of sampling error on the results of Target-rotation. The method is termed oblique Mean-Target-rotation (OMT) and may also be of interest when a few substantial cross-loadings occur in the population because it avoids minimizing the single cross-loadings. Thereby, OMT could be helpful for the investigation of departures from the ICM.

The ICM is a relevant model for the investigation of oblique factor rotation because most researchers will consider a model with minimal cross-loadings as an advantage for factor interpretation. Nevertheless, it might be of interest to compare OMT- and OT-rotation for other population factor models. However, more complex factor models will typically not allow to draw clear conclusions on optimal factor rotation and optimal factor inter-correlations. The reason is that for several more complex models some researchers might prefer larger cross-loadings combined with smaller factor loadings and others might prefer smaller cross-loadings combined with larger factor inter-correlations (Schmitt and Sass, 2011). There is typically no objective way to decide between these preferences.

Effects of sampling error on cross-loadings can be positive and negative. Therefore, the absolute size of a single population cross-loading can be over- or underestimated when sampling error occurs. It is, however, rather unlikely that sampling error affects all cross-loadings on one factor in the same direction. An approximately symmetric distribution of positive and negative effects of sampling error on the cross-loadings on one factor is most likely. One might therefore expect that the positive and negative effects of sampling error on cross-loadings cancel out across cross-loadings so that the mean of the cross-loadings on one factor could be an estimate of the average population cross-loadings on this factor. However, different population factor inter-correlations may result in different population cross-loadings in the corresponding orthogonal loading pattern.

An example for the effect of the correlation between two factors on the cross-loadings of the corresponding orthogonal factors is shown in Table 1. In order to show the effect of sampling error on cross-loadings, the orthogonal population loading pattern is given together with a corresponding orthogonal sample loading pattern for *n* = 1,000 cases. There are two blocks of non-zero cross-loadings in the population loading pattern and, in the sample, the cross-loadings are slightly smaller or larger than the corresponding population cross-loading. The average of cross-loadings for each block of salient loadings will be close to the population cross-loadings. Therefore, the block-wise average of cross-loadings might minimize the effect of sampling error on cross-loadings while it maintains the population mean cross-loading that might be important for factor rotation. The example also illustrates that the larger cross-loadings are eliminated in the oblique loading pattern.

**Table 1:** Example for the effect of sampling error on cross-loadings.

	Population orthogonal loadings			Sample orthogonal loadings (*n* = 1,000)			Sample oblique loadings (*n* = 1,000)		
variables	F1	F2	F3	F1	F2	F3	F1	F2	F3
x_1_	**0.48**	*0.13*	0.00	**0.45**	*0.13*	0.02	**0.46**	0.03	0.03
x_2_	**0.48**	*0.13*	0.00	**0.55**	*0.08*	0.00	**0.58**	−0.05	0.01
x_3_	**0.48**	*0.13*	0.00	**0.43**	*0.12*	−0.04	**0.44**	0.03	−0.04
x_4_	*0.13*	**0.48**	0.00	*0.15*	**0.51**	0.07	0.03	**0.52**	0.04
x_5_	*0.13*	**0.48**	0.00	*0.12*	**0.44**	−0.01	0.02	**0.44**	−0.03
x_6_	*0.13*	**0.48**	0.00	*0.09*	**0.54**	0.01	−0.04	**0.56**	−0.01
x_7_	0.00	0.00	**0.50**	−0.04	0.04	**0.51**	−0.04	0.03	**0.51**
x_8_	0.00	0.00	**0.50**	−0.01	0.03	**0.52**	0.00	0.00	**0.52**
x_9_	0.00	0.00	**0.50**	0.02	−0.01	**0.52**	0.04	−0.04	**0.52**
Factor inter-correlations									
F1	1.00			1.00			1.00		
F2	0.00	1.00		0.00	1.00		0.43	1.00	
F3	0.00	0.00	1.00	0.00	0.00	1.00	−0.02	0.09	1.00

Salient loadings are given in bold face. Large cross-loadings are given in italics.

When the effect of sampling error on cross-loadings is minimized by block-wise averaging and the effect of population cross-loadings is minimized by oblique rotation, the resulting loading patterns with approximately zero-mean cross-loadings might allow for a rather simple interpretation of the factors. For these models, there is no, or when the sum of positive loadings is not perfectly equal to the size of negative loadings, a rather small trade-off between minimizing the absolute size of the cross-loadings and allowing for larger factor inter-correlations. Thus, like the ICM, zero-mean cross-loading models (ZCLM) refer to rather clearly defined oblique rotations of the factors. Therefore, both models are appropriate starting points for the investigation of the effect of sampling error on OMT- and OT-rotation. From a more general perspective, the ICM and the ZCLM can both be regarded as different ways to achieve simple structure. However, loading patterns with other structures may also be of interest (Browne, 2001; Ertel, 2011). The principle of using block-wise mean loadings for target-rotation might also be an interesting option for rotations that are not based on simple structure. But this more general issue is beyond the scope of the present study.

It was expected that the effect of sampling error on the weighted mean cross-loadings computed in OMT-rotation is smaller than on the single cross-loadings used in OT-rotation. Since smaller sample sizes result in larger standard errors of factor loadings, OMT-rotation should be more appropriate for the investigation of small sample sizes than OT-rotation. The standard errors of factor loadings depend on the model estimation method, on the method of factor rotation, and on the complexity of the loading pattern (Zhang, 2014; Zhang and Preacher, 2015). Therefore, several numerical methods like, for example, the nonparametric bootstrap have been proposed for this issue (Zhang, 2014). The exploration of different methods for the computation of standard errors of loadings is beyond the scope of the present study. However, the comparison of the standard deviations of OT- and OMT-rotated loadings in a simulation study should reveal whether averaging cross-loadings minimizes the effect of sampling error on factor loadings. If averaging cross-loadings reduces the effect of sampling error, the standard deviations of OMT-rotated loadings should be smaller than the standard deviations of OT-rotated loadings.

After some definitions, the OMT-rotation and a population example will be presented. A simulation study was performed for the oblique ICM to compare OMT-rotation with conventional oblique Target-rotation (OT). Moreover, OMT- and OT-rotation were compared by means of an empirical example. Finally, recommendations for analyses of oblique ICM and ZCLM by means of Target-rotations are discussed.

## 2 Definitions

According to the population common factor model a random vector x of *p* observed variables is explained by a random vector ξ of *q* common factors and a random vector δ of *p* unique factors. This can be written as

(1) x=Λξ+δ,

where **Λ** is the *p* × *q* matrix of factor loadings and $E\left(\xi \xi '\right)=\Phi ,\;\;diag(\Phi )=I,\;\;E\left(\delta \delta '\right)=\Psi^{2}=diag\left(\Psi^{2}\right),$ and $E\left(\xi \delta \right)=0$ . This implies

(2) $$E\left({\mathrm{xx}}^{'}\right)=\Sigma =\Lambda \Phi \Lambda^{'}+\Psi^{2}=\Lambda_{u}\Lambda_{u}^{'}+\Psi^{2},$$

where **Λ**_u_ is the matrix of common factor loadings for uncorrelated factors, i.e., for Φ = I. Oblique target-rotations (Hurley and Cattell, 1962; Browne, 1972) start from an orthogonal loading matrix **Λ**_u_, which is mostly the unrotated loading matrix resulting from factor extraction.

## 3 Oblique mean-target-rotation

OMT-rotation starts with an orthogonal Target-rotation (Schönemann, 1966) of the unrotated loadings **Λ**_u_ toward a loading Target-matrix **Λ**_T_ corresponding to a perfect ICM, with

(3) $$\Lambda_{T}=\left(I_{q}⊗\;1_{p/q}\right),$$

Where I*_q_* is a *q* × *q* identity matrix, 1_*p/q*_ is a *p/q* × 1 unit-vector representing the Target-loadings and “ ⊗ ” denotes the Kronecker-product. The resulting **Λ**_1_ represents an orthogonal loading matrix where the salient loadings are a least square approximation of Λ_T_. Weighted mean loadings are computed for each block of salient loadings

(4) $$\Lambda_{1m}={\left(\Lambda_{1}\cdot \Lambda_{T}\right)}^{'}\Lambda_{1}{\left({\left(\Lambda_{1}\cdot \Lambda_{T}\right)}^{'}\Lambda_{T}\right)}^{-1},$$

where “ ⋅ ” is the Hadamard-product. Therefore, Λ_1_ ⋅ Λ_T_ yields the weights of the salient loadings so that the cross-loadings are weighted by the salient-loadings of the respective variable on the respective factor. The resulting weighted mean loading matrix Λ_1m_ is a *q* × *q* matrix so that a *q* × *q* identity matrix I*_q_* can be used as Target-matrix for oblique Target-rotation according to Hurley and Cattell (1962), where the transformation matrix

(5) $$T={\left(\Lambda_{1m}^{'}\Lambda_{1m}\right)}^{-1}\Lambda_{1m}I_{q},$$

is normalized in order to get

(6) $$T_{n}=diag{\left(T{}^{'}T\right)}^{-0.5}T.$$

This transformation matrix is then used for rotation of the complete loadings, with the reference structure

(7) $$\Lambda_{2}=\Lambda_{1}T_{n},$$

and the OMT-rotated loading pattern

(8) $$\Lambda_{O}=\Lambda_{2}diag{\left({\left(T_{n}^{'}T_{n}\right)}^{-1}\right)}^{0.5},$$

and the OMT-rotated factor inter-correlations

(9) $$\Phi_{O}={\left(\Lambda_{O}^{'}\Lambda_{O}\right)}^{-1}\Lambda_{O}^{'}\left(\Lambda_{u}\Lambda_{u}^{'}\right)\Lambda_{O}{\left(\Lambda_{O}^{'}\Lambda_{O}\right)}^{-1}.$$

In order to evaluate whether $\Lambda_{1m}^{'}\Lambda_{1m}$ is ill-conditioned, the condition-number κ is computed (Moler, 2008). If κ is large, the inversion of the matrix may lead to numerical imprecision. As in ridge regression, there is the option to add small ridge constants when κ is large and to retain the solution with the largest mean congruence (Tucker, 1951) of **Λ**_O_ with **Λ**_T_. For large sample sizes and large salient loadings, this option might be irrelevant, but in general, this option could not be harmful as the solution with the best congruence with **Λ**_T_ is retained. The loop for the ridge constant can be found in the R- and SPSS-script in the Supplementary Material.

## 4 Population example

An R-script as well as an SPSS-script based on the example presented here, allowing for OMT and OT-rotation is given in the Supplementary Material. Users of the R-script may install R-4.3.1 and replace the initial orthogonal loadings by orthogonal loadings of interest. The following orthogonal loading matrix shows the difference between OMT- and OT-rotation (see Table 2, left). As the mean of the cross-loadings that balance out within each block of salient loadings is zero within each block of salient loadings, the ideal OMT-rotated loading pattern is already reached so that the initial orthogonal solution is not modified by OMT-rotation. In contrast, OT-rotation minimized the negative loadings and thereby introduces a negative factor inter-correlation (Table 2, bottom). In consequence, the block-wise mean cross-loadings of the OT-rotated solution is not zero. It is, of course, a matter of theoretical preference, which model should be used. However, it is clear that the OMT-rotated solution could also be of interest when the mean non-salient loadings are expected to be zero.

**Table 2:** Population example with initial orthogonal loadings.

	Initial orthogonal loadings			OT-rotated loadings			OMT-rotated loadings		
Variables	F1	F2	F3	F1	F2	F3	F1	F2	F3
x_1_	**0.50**	0.20	−0.20	**0.52**	0.25	−0.11	**0.50**	0.20	−0.20
x_2_	**0.50**	−0.20	0.20	**0.52**	−0.11	0.25	**0.50**	−0.20	0.20
x_3_	**0.50**	0.20	−0.20	**0.52**	0.25	−0.11	**0.50**	0.20	−0.20
x_4_	**0.50**	−0.20	0.20	**0.52**	−0.11	0.25	**0.50**	−0.20	0.20
x_5_	**0.50**	0.20	−0.20	**0.52**	0.25	−0.11	**0.50**	0.20	−0.20
x_6_	**0.50**	−0.20	0.20	**0.52**	−0.11	0.25	**0.50**	−0.20	0.20
x_7_	0.20	**0.50**	−0.20	0.25	**0.52**	−0.11	0.20	**0.50**	−0.20
x_8_	−0.20	**0.50**	0.20	−0.11	**0.52**	0.25	−0.20	**0.50**	0.20
x_9_	0.20	**0.50**	−0.20	0.25	**0.52**	−0.11	0.20	**0.50**	−0.20
x_10_	−0.20	**0.50**	0.20	−0.11	**0.52**	0.25	−0.20	**0.50**	0.20
x_11_	0.20	**0.50**	−0.20	0.25	**0.52**	−0.11	0.20	**0.50**	−0.20
x_12_	−0.20	**0.50**	0.20	−0.11	**0.52**	0.25	−0.20	**0.50**	0.20
x_13_	0.20	−0.20	**0.50**	0.25	−0.11	**0.52**	0.20	−0.20	**0.50**
x_14_	−0.20	0.20	**0.50**	−0.11	0.25	**0.52**	−0.20	0.20	**0.50**
x_15_	0.20	−0.20	**0.50**	0.25	−0.11	**0.52**	0.20	−0.20	**0.50**
x_16_	−0.20	0.20	**0.50**	−0.11	0.25	**0.52**	−0.20	0.20	**0.50**
x_17_	0.20	−0.20	**0.50**	0.25	−0.11	**0.52**	0.20	−0.20	**0.50**
x_18_	−0.20	0.20	**0.50**	−0.11	0.25	**0.52**	−0.20	0.20	**0.50**
Factor inter-correlations									
F1	1.00			1.00			1.00		
F2	0.00	1.00		−0.22	1.00		0.00	1.00	
F3	0.00	0.00	1.00	−0.22	−0.22	1.00	0.00	0.00	1.00

Salient loadings are given in bold face.

## 5 Simulation study

### 5.1 Specification

#### 5.1.1 Independent variables

A simulation study based on the population ICM and population ZCLM with *q* ∈ {3, 6, 9, 12} factors and *p/q* ∈ {5, 8} salient loadings per factor was performed. For *p/q* = 5 two levels of salient loadings were introduced with

(10) $$\lambda_{.50}=\;\left[\begin{array}{c} .40\\ \begin{array}{l} .45\\ .50 \end{array}\\ .55\\ .60 \end{array}\right]\;\;and\;\lambda_{.70}=\;\left[\begin{array}{c} .60\\ \begin{array}{l} .65\\ .70 \end{array}\\ .75\\ .80 \end{array}\right]$$

for each salient loading block with *p/q* = 5. For *p/q* = 8 the two levels of salient loadings were

(11) $$\lambda_{.50}=\;\left[\begin{array}{l} \begin{array}{c} .38\\ \begin{array}{l} .42\\ .45 \end{array}\\ .48\\ .52 \end{array}\\ .55\\ .58\\ .62 \end{array}\right]\;\;and\;\lambda_{.70}=\;\left[\begin{array}{c} .58\\ \begin{array}{l} .62\\ .65 \end{array}\\ .68\\ \begin{array}{l} .72\\ .75\\ .78\\ .82 \end{array} \end{array}\right].$$

The standard deviation of the salient loadings was about 0.08 for both levels of *p/q*. The ICM was based on zero population cross-loadings (*CL* = 0) and the ZCLM was based on a condition with a balanced set of non-zero population cross-loadings (*CL* ≠ 0). In the condition with non-zero cross-loadings the largest absolute population cross-loadings were one third of the average population salient loading. As an example, the non-zero population cross-loadings for *p/q* = 5 were

(12) $$CL_{.50}=\;\left[\begin{array}{c} \;\;.17\\ \begin{array}{l} -.08\\ \;\;.06 \end{array}\\ -.04\\ \;\;.03 \end{array}\right]\;\;\mathrm{and}\;CL_{.70}=\;\left[\begin{array}{c} \;\;.23\\ \begin{array}{l} -.12\\ \;\;.08 \end{array}\\ -.06\\ \;\;.05 \end{array}\right].$$

**Figure 1:**
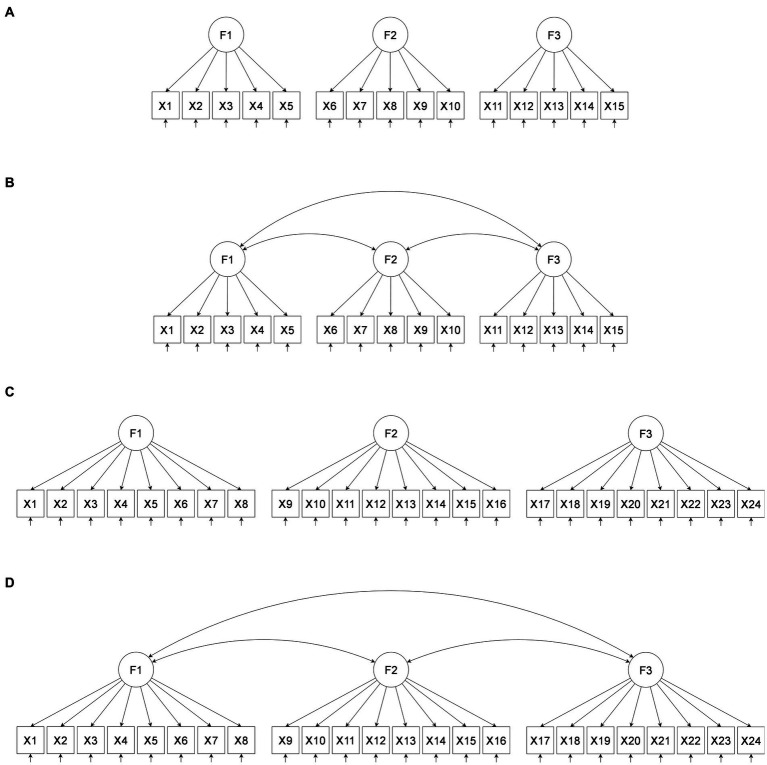
Example for models with three factors (*q* = 3) and *CL* = 0, **(A)** for *ϕ* = 0.00 and *p/q* = 5, **(B)** for *ϕ* > 0.00 and *p/q* = 5, **(C)** for *ϕ* = 0.00 and *p/q* = 8, **(D)** for *ϕ* > 0.00 and *p/q* = 8; *q* indicates the number of factors; *p/q* indicates the number of salient loadings per factor.

The loadings in *CL*_.50_ have *M* = −0.03 and *SD* = 0.10 and for the loadings in *CL*_.70_ have *M* = −0.04 and *SD* = 0.14. Small deviations from zero-mean were defined in order to provide a realistic approximation to the ZCLM. The columns denoted as *CL*_.50_ and *CL*_.70_ can be inserted into the loading pattern as presented here, or the columns may be multiplied by −1. This results in different patterns of zero-mean non-zero cross-loadings. In order to cover a large number of combinations of cross-loadings, the patterns of cross-loading columns were slightly different for *p/q* = 5 and *p/q* = 8. As OMT-rotation is based on mean cross-loadings, which are zero for the columns, OMT-rotation should be less affected by the different loading patterns than OT-rotation. Three levels of population factor inter-correlations *ϕ* ∈ {0.00, 0.25, 0.50} and five sample sizes *n* ∈ {100, 150, 200, 300, 500} were investigated. The combinations of independent variables result in 4 (*q*) × 2 (*p/q*) × 2 (λ_.50_, λ_.70_) × 2 (*CL* = 0, *CL* ≠ 0) × 3 (*ϕ*) × 5 (*n*) = 480 conditions of the simulation study. An example for the *q* = 3 and *CL* = 0, for *ϕ* = 0.00 and *ϕ* > 0.00 is presented in Figure 1. The first panel (A) of Figure 1 shows the condition based on the ICM (*CL* = 0) with *q* = 3 factors, zero factor inter-correlations (*ϕ* = 0.00) and five variables with salient loadings on each factor (*p/q* = 5). The second panel (B) shows the same ICM with non-zero factor inter-correlations (*ϕ* > 0.00). This refers to the two levels of factor inter-correlations that were included into the study (*ϕ* = 0.25 and *ϕ* = 0.50). The third panel (C) shows the uncorrelated ICM with *q* = 3 based on eight loadings with salient loadings on each factor (*p/q* = 8), and the fourth panel (D) shows the correlated ICM with *q* = 3 based on *p/q* = 8. That is, the last panel refers to the conditions based on *ϕ* = 0.25 and *ϕ* = 0.50.

The independent variables of the simulation study are summarized in Table 3.

**Table 3:** Independent variables of the simulation study.

Independent variables	Abbreviation (number of levels)	levels
Factor rotation method	*Rot (2)*	OT- vs. OMT-rotation
Number of factors	*q (4)*	3, 6, 9, 12
Number of salient loadings	*p/q* (2)	5, 8
Salient loading size	λ (2)	0.50, 0.70
Cross-loadings	*CL* (2)	zero vs. non-zero
Factor inter-correlations	*ϕ* (3)	0.00, 0.25, 0.50
Sample size	*n* (5)	100, 150, 200, 300, 500

#### 5.1.2 Dependent variables

The dependent variables were the OT- and OMT-factor inter-correlations and their bias which was computed as the difference between the OT/OMT-factor inter-correlations and the corresponding population factor inter-correlations (*ϕ*). Moreover, the root mean square (RMS) difference of the OT- and OMT-rotated factor pattern with the population loading pattern as well as the recovery of factor scores, i.e., factor score indeterminacy (Grice, 2001), the correlation of the regression factor scores calculated from the OT/OMT-rotated factors with the true factors, was computed.

#### 5.1.3 Data generation

Data generation was performed with the R-package ‘fungible’ provided by Waller et al. (2023) based on Waller (2016), where population loadings and factor inter-correlations were entered in order to generate sample correlation matrices. The population factor models were entered into the ‘MonteCarlo’ option of the ‘simFA’ package and correlation matrices were generated for continuous variables under multivariate normality. For each of the 480 conditions 1,000 sample correlation matrices were generated. Least squares factor analysis with the correct number of factors was performed with the ‘simFA’ package and unrotated factor loadings were computed. The unrotated factor loadings were entered into the script as it can be found in the Supplement (p. 7) to compute the OT- and OMT-rotated loadings and the corresponding factor inter-correlations. To compare the correlation of the factor score predictor with the original factor for OT- and OMT-rotated factors (factor score indeterminacy), we used the ‘FactorScores’ option. As the computation of individual scores needs a considerable amount of computation time, we restricted this analysis to a few conditions based on *n* = 100 where large differences between rotation methods can be expected. Therefore, factor scores were investigated for the condition with *n* = 100, *q* = 6 and *q* = 9, *p/q* = 5, *ϕ = 0.25,* λ = 0.50, *CL* = 0 and *CL* ≠ 0.

### 5.2 Results

Repeated measures ANOVA was performed for bias of factor inter-correlations as dependent variable and OT- versus OMT-rotation as within-factor (Rot) and number of factors (*q*), number of salient loadings (*p/q*), loading size (λ), cross-loadings (*CL*), factor inter-correlations (*ϕ*), and sample size (*n*) as between-factors. All effects were significant at *p* < 0.001 and the corresponding effect sizes $\eta_{p}^{2}$ are reported in Table 4. We followed Skrondal’s (2000) recommendation of reporting only main effects and two-way interaction effects on each dependent variable of Monte Carlo experiments to facilitate interpretation.

**Table 4:** Repeated measures ANOVA main effects and two-way interactions for the conditions of the simulation study (independent variables) and bias of factor inter-correlations (dependent variable) based on OT- and OMT-rotation.

Within/between effects	*F*	*df*	$\eta_{p}^{2}$	Between effects	*F*	*df*	$\eta_{p}^{2}$
Rotation	9259.92	1	0.02	*q*	5329.83	3	0.03
Rotation *× q*	29703.17	3	0.16	*ϕ*	141376.72	2	**0.37**
Rotation *× ϕ*	209911.87	2	**0.47**	λ	135796.55	1	0.22
Rotation *×* λ	191336.81	1	0.29	*n*	31880.46	4	0.21
Rotation *× n*	31674.43	4	0.21	*CL*	245893.48	1	**0.34**
Rotation *× CL*	519432.14	1	**0.52**	*pq*	200733.39	1	0.30
Rotation *× p/q*	298812.24	1	**0.38**	*q* × *ϕ*	10588.39	6	0.12
				*q* × **λ**	8867.27	3	0.05
				*q* × *n*	2090.47	12	0.05
				*q* × *CL*	29037.07	3	0.15
				*q* × *pq*	16967.97	3	0.10
				**λ** × *ϕ*	60445.83	2	0.20
				*ϕ* × *n*	11725.43	8	0.16
				*CL* × *ϕ*	7259.88	2	0.03
				*pq* × *ϕ*	8401.49	2	0.03
				**λ** × *n*	14728.05	4	0.11
				**λ** × *CL*	6979.38	1	0.01
				*pq* × **λ**	4950.14	1	0.01
				*CL* × *n*	91.83	4	0.00
				*pq* × *n*	90.93	4	0.00
				*pq* × *CL*	133206.31	1	0.22

All effects were significant at *p* < 0.001; $\eta_{p}^{2}$ > 0.30 are given in bold-face.

The largest effect occurred for the interaction of rotation method with cross-loadings. Whereas the negative bias of OT-rotation for factor inter-correlations was larger (*M* = −0.07; *SE* < 0.001) than the negative bias of OMT-rotation (*M* = −0.02; *SE* < 0.001) for *CL* = 0 (ICM), the positive bias of OT-rotation was larger (*M* = 0.04; *SE* < 0.001) than the positive bias of OMT-rotation (*M* < 0.001; *SE* < 0.001) for *CL* ≠ 0 (ZCLM). The extremely small standard errors indicate that the mean differences are significant. The second largest effect occurred for the interaction of rotation method with factor inter-correlations. Whereas the bias of OT-rotation on factor inter-correlations was more positive (*M* = 0.04; *SE* < 0.001) than the bias of OMT-rotation (*M* = 0.002; *SE* < 0.001) for *ϕ =* 0.00, the bias for *ϕ =* 0.25 was close to zero for OT-rotation (*M* = 0.004; *SE* < 0.001) and for OMT-rotation (*M* < 0.001; *SE* < 0.001), and for *ϕ =* 0.50, it was more negative for OT-rotation (*M* = −0.09; *SE* < 0.001) than for OMT-rotation (*M* = −0.03; *SE* < 0.001). The next largest effect was the interaction of rotation method with the number of salient loadings per factor (*p/q*). For *p/q* = 5 the negative bias of OT-rotation (*M* = −0.06; *SE* < 0.001) was more substantial than the negative bias of OMT-rotation (*M* = −0.02; *SE* < 0.001) and for *p/q* = 8 the positive bias was larger for OT-rotation (*M* = 0.03; *SE* < 0.001) than for OMT-rotation (*M* = 0.003; *SE* < 0.001). These largest interaction effects indicate that the positive and negative biases that are related to the conditions *CL*, *ϕ*, and *p/q* were larger for OT-rotation than for OMT-rotation.

**Figure 2:**
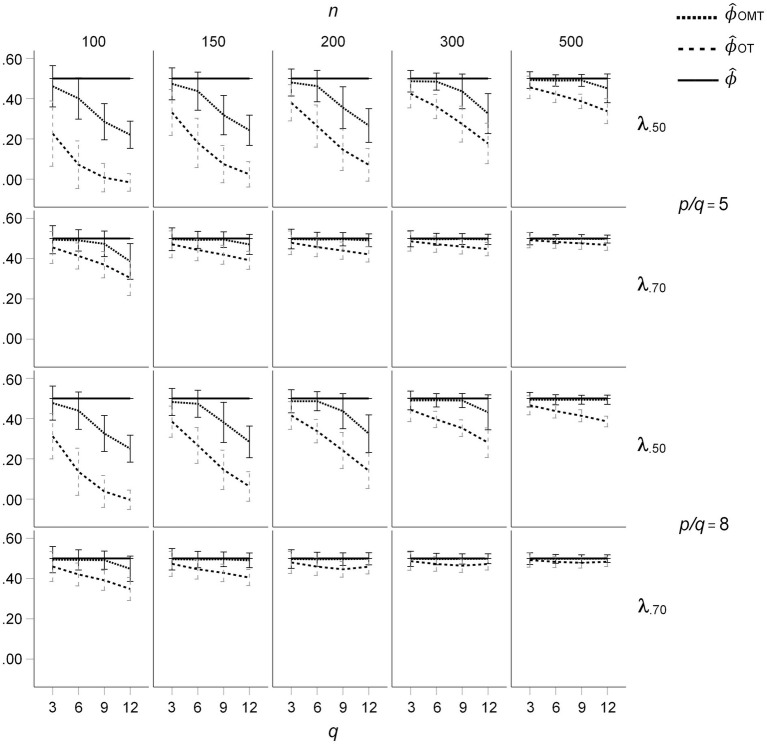
Means and standard deviations of inter-factor correlations resulting from OT- and OMT-rotation for the ICM, population factor inter-correlations of *ϕ* = 0.50; *q* indicates the number of factors.

**Figure 3:**
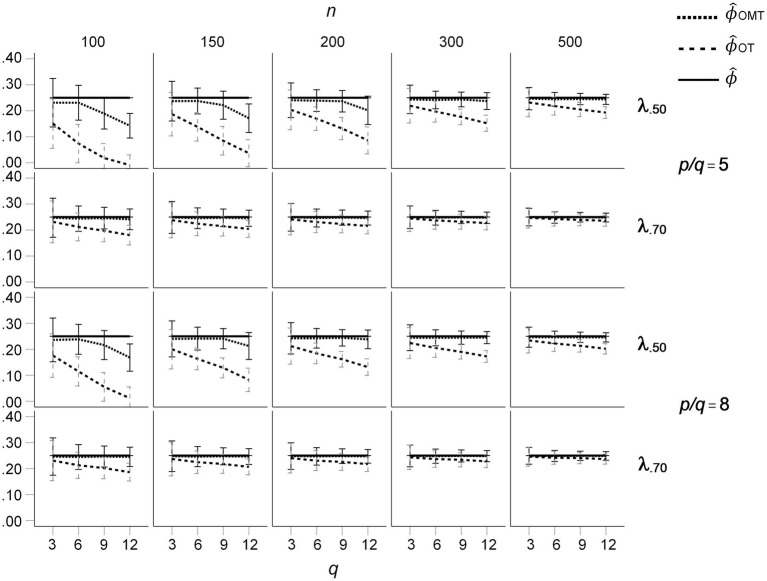
Means and standard deviations of inter-factor correlations resulting from OT- and OMT-rotation for the ICM, population factor inter-correlations of *ϕ* = 0.25; *q* indicates the number of factors.

The descriptive results for the factor inter-correlations for *CL* = 0 (ICM) based on population factor inter-correlations of *ϕ* = 0.50 are presented in Figure 2. For mean salient loadings of 0.50 and samples of *n* = 200 and below, the mean inter-correlations of OT-rotated factors are considerably smaller than *ϕ* = 0.50. In contrast, the mean inter-correlations of the OMT-rotated factors are much closer to 0.50 and show a smaller negative bias. For *q* = 12 factors and mean salient loadings of 0.50, the mean inter-correlations of the OT-rotated factors are zero, whereas the mean inter-correlations of the OMT-rotated factors are a bit larger than 0.20. Thus, the under-estimation of the inter-correlations is present in all target-rotated factors but it is much smaller for the OMT-rotated factors than for the OT-rotated factors. The under-estimation of the population factor inter-correlations is considerably reduced for mean salient loadings of 0.70 (see Figure 2). The under-estimation of factor inter-correlations was also smaller for OMT-rotated factors than for OT-rotated factor for *ϕ* = 0.25 (see Figure 3). Overall, the size of the effects was reduced and the pattern was the same as for *ϕ* = 0.50. No under-estimation of the population factor inter-correlations and no substantial difference between OT- and OMT-rotated factors occurred for *ϕ* = 0.00 (see Supplementary Figure S1). However, in this condition, the standard deviation of the factor inter-correlations was larger for OT-rotated factors than for OMT-rotated factors for mean salient loadings of 0.50, *n* = 100, and *q* = 12.

**Figure 4:**
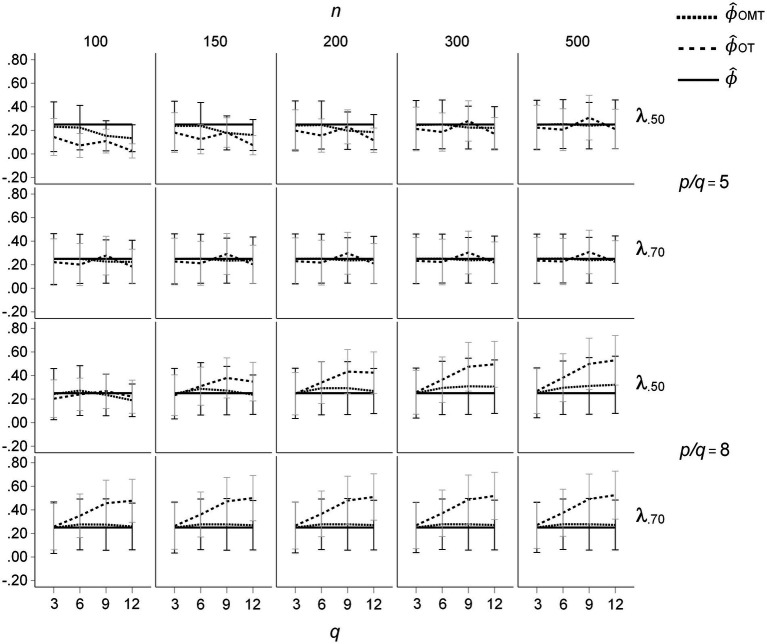
Means and standard deviations of inter-factor correlations resulting from OT- and OMT-rotation for the ZCLM, population factor inter-correlations of *ϕ* = 0.50; *q* indicates the number of factors.

Whereas the results for the ICM show that the under-estimation of factor inter-correlations for OT-rotation is more pronounced than for OMT-rotation, the results for the ZCLM are more complex (see Figure 4). For *p/q* = 5, there are small over- and underestimations of the inter-correlations of the OT-rotated factors for different numbers of factors, whereas the inter-correlations of the OMT-rotated factors remain rather similar across different numbers of factors. In contrast, for *p/q* = 8, an over-estimation of factor inter-correlations for OT-rotated factors increases with the number of factors. Again, only very small variations of the inter-correlations of the OMT-rotated factors occurred. Although the different patterns of non-zero population cross-loadings induce different factor inter-correlations of OT-rotated factors, the inter-correlation of the OMT-rotated factors is only slightly affected by the different patterns.

**Figure 5:**
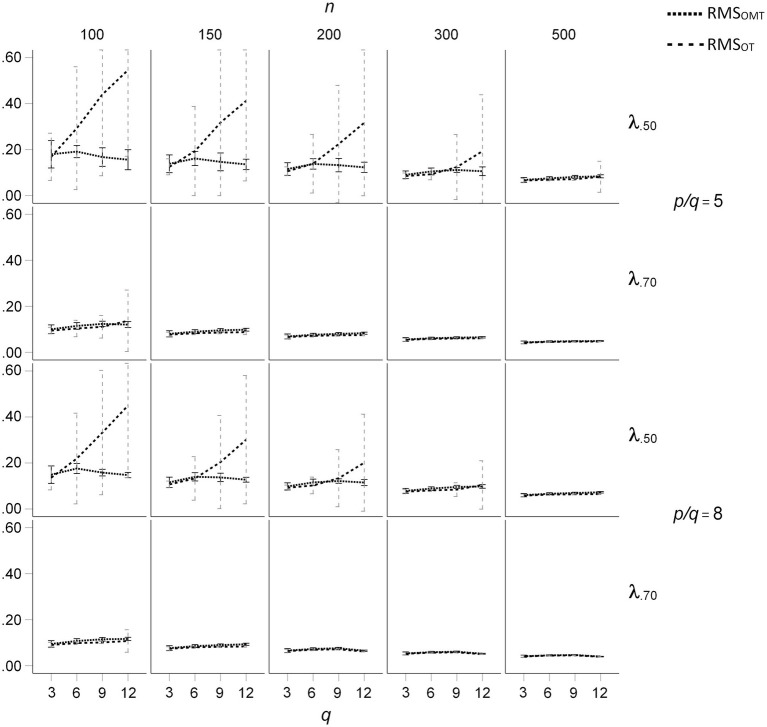
Root Mean Square (RMS) difference between the population loading pattern and the OT- and OMT-rotated loading patterns for population factor inter-correlations of *ϕ* = 0.50; *q* indicates the number of factors.

**Figure 6:**
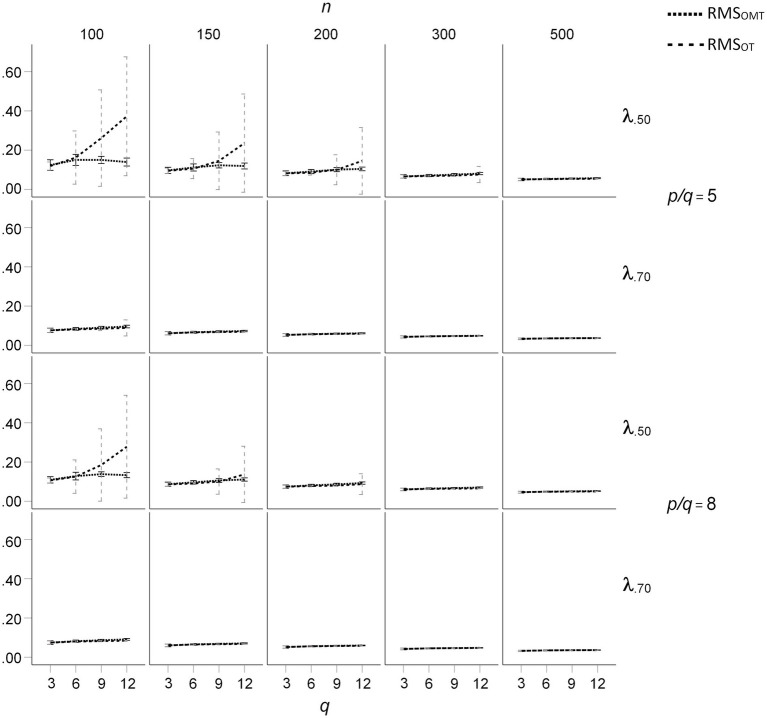
Root Mean Square (RMS) difference between the population loading pattern and the OT- and OMT-rotated loading patterns for population factor inter-correlations of *ϕ* = 0.25; *q* indicates the number of factors.

The mean RMS differences of the OT- and OMT-rotated loading patterns with the population loadings for the *ϕ* = 0.50 condition are presented in Figure 5. For all loading sizes and sample sizes, the mean RMS differences were nearly the same for *q* = 3. For *q* > 6, mean salient loadings of 0.50, and sample sizes smaller than 300, the mean RMS differences were substantially larger for OT-rotated factor patterns than for OMT-rotated factor patterns. In these conditions, the mean RMS differences increased with *q* for the OT-rotated factor patterns, whereas they did not substantially increase with *q* for the OMT-rotated factor patterns. In these conditions, the standard deviations of the RMS differences were much larger for the OT-rotated factor patterns than for the OT-rotated factor patterns (see Figure 5). For *ϕ* = 0.25 the effects of *q*, *n*, and mean salient loading size on mean RMS differences were smaller than for *ϕ* = 0.50, but the pattern of results was the same (see Figure 6). For *ϕ* = 0.00 the mean RMS differences were very small and only a small increase of mean RMS differences occurred for OT-rotated factors for *n* = 100, *q* > 6, and mean salient loadings of 0.50.

Differences of OT- and OMT-rotation for the standard deviations of salient loadings and cross-loadings are presented for *n* = 100 and *p/q* = 5 in the Supplementary Figures S3A–D. In this condition, the largest standard deviations of loadings can be expected. For the ICM, the standard deviations of OT-rotated cross-loadings were considerably larger than the standard deviations of the OMT-rotated loadings for *q* ≥ 6 at all levels of *ϕ* (Supplementary Figures S3A,B). For the ZCLM, considerably larger standard deviations of OT-rotated loadings occurred for *q* = 6 and *ϕ* = 0.50 (Supplementary Figure S3C) and for *q* > 6 at all levels of *ϕ*. For *q = 3*, the standard deviations of OT-rotated and OMT-rotated loadings were similar for all levels of *ϕ* (Supplementary Figure S3D).

Repeated measures ANOVA was performed for factor score indeterminacy of OT-rotated and OMT-rotated factors. Rotation method was a within-factor (Rot), and number of factors (*q*), and cross-loadings (*CL*) were between-factors. The main effect of rotation method and the interactions of rotation method with *q* and *CL* were significant at *p* < 0.001 and the corresponding effect sizes $\eta_{p}^{2}$ are reported in Table 5. The largest effect occurred for rotation method with *M* = 0.52 (*SE* = 0.002) for OT-rotation and *M* = 0.60 (*SE* = 0.001) for OMT-rotation. The second largest effect was the interaction of rotation method with the number of factors, indicating that the difference between the rotation methods was smaller for *q* = 6 (OT-rotation: *M* = 0.58, *SE* = 0.003; OMT-rotation: *M* = 0.63, *SE* = 0.001) than for *q* = 9 (OT-rotation: *M* = 0.46, SE = 0.003; OMT-rotation: *M* = 0.57, SE = 0.001). The remaining effect sizes were rather small.

**Table 5:** Repeated measures ANOVA results for the conditions of the simulation study (independent variables) and indeterminacy of factors based on OT- and OMT-rotation.

Within/between effects	*F*	*df*	$\eta_{p}^{2}$
Rotation	3544.54	1	**0.47**
Rotation *× q*	495.86	1	0.11
Rotation *× CL*	265.69	1	0.06
Rotation *× q × CL*	61.71	1	0.02

All reported effects were significant at *p* < 0.001; $\eta_{p}^{2}$ > 0.30 are given in bold-face.

## 6 Empirical example

As an empirical example a subsample of participants responses to 25 items from the Open-Source Psychometrics Project^1^ based on the Big-Five Factor Markers (BIG5.zip, last updated 5/18/2014, retrieved on 08/22/2023) from the International Personality Item Pool (IPIP, Goldberg, 1992) was used. Only the first 19,700 participants (age/years: *M* = 26.27, *SD* = 11.59; gender: 11,973 females, 7,601 males, 102 others, 24 missing values) from the total file of 19,719 participants were used in order to split the total sample into 197 subsamples each containing the responses of 100 participants to the first four items (E1-E4, N1-N4, A1-A4, C1-C4, O1-O4) of each of the five factors. Only a subsample of items was used in order to investigation a data set that is less favorable for optimal factor rotation.

The aim was to compare the OT- and OMT-rotated five-factor solution of the total sample with the OT- and OMT-rotated five-factor solutions of the subsamples. Principal axis factoring of the total sample and of the subsamples was performed with IBM SPSS Version 29.0 and OT- and OMT-rotation was performed with the code provided in the Supplementary Material. The rotated solutions for the total sample are presented in Table 5. The OT- and OMT-rotated loading patterns are very similar which indicates that for the very large total sample both rotation methods work well. The inter-correlations of the OMT-rotated factors were a bit larger than the inter-correlations of the OT-rotated factors (Table 6).

**Table 6:** OT- and OMT-rotated five factor loading patterns and factor inter-correlations for 20 BIG-Five Markers of the total sample.

	OMT-rotation					OT-rotation				
	E	N	A	C	O	E	N	A	C	O
E1	**0.71**	0.03	−0.12	−0.06	−0.01	**0.66**	−0.03	−0.02	−0.01	0.01
E2	**0.75**	0.15	−0.10	−0.07	0.03	**0.69**	0.09	0.01	−0.01	0.05
E3	**0.65**	−0.13	0.14	0.06	−0.06	**0.62**	−0.19	0.23	0.09	−0.04
E4	**0.77**	0.04	−0.15	0.03	0.02	**0.72**	−0.02	−0.05	0.08	0.04
N1	0.02	**0.76**	−0.01	−0.02	−0.04	0.00	**0.75**	−0.01	−0.02	−0.06
N2	−0.02	**0.60**	−0.10	0.08	0.00	−0.03	**0.60**	−0.11	0.09	−0.01
N3	−0.02	**0.72**	0.09	0.04	0.00	−0.03	**0.72**	0.08	0.03	−0.01
N4	−0.09	**0.34**	−0.01	−0.05	0.12	−0.10	**0.34**	−0.02	−0.04	0.11
A1	0.03	0.05	**0.39**	0.00	0.11	0.03	0.04	**0.40**	0.00	0.12
A2	0.34	0.03	**0.43**	−0.08	0.05	0.32	0.00	**0.48**	−0.07	0.06
A3	−0.17	−0.16	**0.45**	0.22	−0.07	−0.14	−0.15	**0.42**	0.19	−0.06
A4	−0.02	0.07	**0.80**	−0.07	−0.04	−0.02	0.05	**0.79**	−0.10	−0.03
C1	0.06	0.04	−0.01	**0.53**	0.04	0.09	0.04	0.00	**0.54**	0.05
C2	−0.05	0.02	−0.06	**0.58**	−0.11	−0.01	0.04	−0.07	**0.57**	−0.10
C3	−0.01	0.12	0.05	**0.36**	0.18	0.01	0.13	0.06	**0.37**	0.18
C4	0.04	−0.16	0.02	**0.66**	−0.04	0.08	−0.15	0.03	**0.66**	−0.02
O1	0.06	0.05	−0.06	0.03	**0.47**	0.06	0.04	−0.03	0.08	**0.46**
O2	−0.01	−0.08	−0.05	0.05	**0.74**	0.00	−0.08	−0.01	0.11	**0.74**
O3	0.04	0.10	0.01	−0.11	**0.38**	0.03	0.09	0.03	−0.07	**0.38**
O4	−0.04	−0.02	0.06	−0.04	**0.64**	−0.03	−0.02	0.08	0.00	**0.63**
	OMT factor inter-correlations					OT factor inter-correlations				
E	1.00					1.00				
N	−0.31	1.00				−0.20	1.00			
A	0.35	0.00	1.00			0.20	0.03	1.00		
C	0.15	−0.24	0.18	1.00		−0.02	−0.25	0.18	1.00	
O	0.11	−0.14	0.16	0.11	1.00	0.04	−0.09	0.08	−0.01	1.00

Salient loadings that are conform to the BIG-Five model are given in bold face.

Overall, 195 out of the 197 principal factor analyses converged. OT- and OMT-rotation was performed for the unrotated factor solutions and the RMS difference of each of the rotated factor patterns with the corresponding rotated factor pattern of the total sample was computed. When for RMS-OT five values greater one were set to one, the mean of RMS-OT was 0.18 (*SD* = 0.19), for RMS-OMT no values greater one occurred and the mean RMS-OMT was 0.16 (*SD* = 0.06). For the factor inter-correlations of the OT-rotated factors RMS-OT was 0.25 (*SD* = 0.21, two values greater one were set to one), for the factor inter-correlations of the OMT-rotated factors RMS-OMT was 0.15 (*SD* = 0.05, no values greater one occurred).

## 7 Discussion

Investigations of simple structure by means of EFA are still relevant, also because analyses of simple structure models by means of confirmatory factor analyses may lead to series of model-modifications. It was, however, expected that sampling error substantially affects results of conventional Target-rotation because single cross-loadings are minimized. In order to reduce the effect of sampling error on results, OMT-rotation was proposed which minimizes mean cross-loadings instead of single cross-loadings. It was shown in a population example that minimizing single cross-loadings by means of conventional OT-rotation may lead to ambiguous results, when the mean cross-loadings are close to zero while the absolute size of the cross-loadings is substantial. In the population model, the observed variables with single cross-loadings that were close to zero after rotation were arbitrary because the variables all had the same absolute cross-loading before rotation. This indicates that OMT-rotation may be of special interest when the cross-loadings with positive and negative sign balance out.

Accordingly, OT- and OMT-rotation were compared in a simulation study based on the oblique ICM, comprising cross-loadings that are exactly zero in the population, and a model with non-zero population cross-loadings resulting in zero-mean cross-loadings (ZCLM) were investigated in a simulation study. ANOVA revealed that the bias of the factor inter-correlations resulting from OT- and OMT-rotation varied for the type of model (ICM vs. ZCLM), the size of the population factor inter-correlations, and the number of variables with salient loadings per factor. For these conditions, the positive and negative biases of the factor inter-correlations were more pronounced for OT-rotation than for OMT-rotation.

ANOVA and descriptive results revealed that, for the oblique ICM, sampling error may induce negative bias to Target-rotated factor inter-correlations. The negative bias of the factor inter-correlations in the ICM was substantially more pronounced for OT-rotation than for OMT-rotation, especially for small sample sizes, moderate mean salient loadings, and a large number of factors. For 12 factors, 100 cases, mean salient loadings of 0.50, and population inter-correlations of 0.50, the mean sample inter-correlations of OT-rotated factors was zero, whereas it was greater 0.20 for OMT-rotated factors. The mean RMS differences of rotated factor patterns and the population factor pattern were larger for OT-rotation than for OMT-rotation. Thus, when samples size was small and the number of factors large, loading patterns and factor inter-correlations were more similar to the population loading patterns and factor inter-correlations for OMT-rotation than for OT-rotation. However, no relevant differences between the rotation methods were found for the uncorrelated ICM. For the ZCLM, more than three factors, and 8 variables with salient loadings per factor, an overestimation of the inter-correlations of the OT-rotated factors occurred that did not occur for the OMT-rotated factors. This reveals that the size and direction of bias of factor inter-correlations does not only depend on sampling error but also on the type of model. Therefore, recommendations regarding the minimum sample size should be based on general studies concerning this issue. A sample size of *n* = 50 has been recommended as a reasonable absolute minimum for exploratory factor analysis (de Winter et al., 2009). However, the study of de Winter et al. (2009) reveals that even smaller samples might be possible when the salient loadings are large, the number of observed variables is large and the number of factors low. As the minimum sample size of the present study was *n* = 100, it remains open for further research whether OMT-rotation may help to improve results of very small samples. Nevertheless, the present simulation study shows that OMT-rotation reduced the standard deviations of loadings, especially when the number of factors is large, and the combined effect of sampling error and model parameters on the bias of factor inter-correlations. Overall, the effect of the conditions of the simulation study on positive and negative bias of factor inter-correlations was more pronounced for OT-rotation than for OMT-rotation. It should be noted that the simulated non-zero population cross-loadings in the ZCLM were not extremely large as they were at maximum one third of the average salient loadings. As small ZCLM cross-loadings resulted in larger bias of factor inter-correlations for OT-rotation than for OMT-rotation, researchers expecting that non-zero cross-loadings cancel out may consider OMT-rotation.

An empirical example was based on open data for the BIG-five model of personality (Goldberg, 1992). A large total sample based on four marker variables per factor was divided into several subsamples based on 100 participants in order to investigate the similarity of the OT- and OMT-rotated subsample solutions with the corresponding OT- and OMT-rotated total sample solutions. The similarity of the rotated loading patterns and factor inter-correlations for the subsamples with the corresponding rotated loading pattern and factor inter-correlations for the total sample was more pronounced for OMT-rotation than for OT-rotation. This indicates that OMT-rotation may help to get more robust results, especially when the number of marker variables per factor and the sample size are rather small.

Overall, the results of the simulation study and of the empirical example indicate that OMT-rotation is more robust than OT-rotation. The robustness refers to effects of sampling error and to the distribution of non-zero cross-loadings across factors. Therefore, OMT-rotation can be recommended when an oblique ICM or a ZCLM is expected and when salient loadings are moderate, factor numbers large, and sample sizes small. The relevant orthogonal/unrotated loading matrices for OMT-rotation may be entered into the R-script or into the SPSS-script provided in the Supplementary Material.

From a broader perspective, it should be noted that less biased factor inter-correlations are an important basis for hierarchical factor models. Factor prediction, as it can be performed with ESEM, also needs optimal estimates of the factor inter-correlations. Further research may consider the investigation of OMT-rotation with very small samples (*n* = 50 and below). Moreover, the basic idea of OMT-rotation, i.e., averaging loadings in order to provide more suitable initial loadings for factor rotation, may be investigated for other methods of factor rotation. The main idea from the present study in this respect is that the transformation matrix for factor rotation derived from rotation of an averaged loading pattern can be used for rotation of the loading pattern based on single observed variables. That is, not only Target-rotation may be improved by reducing the effect of single loadings on the results. The effect of averaging loadings may be beneficial for analytical methods of factor rotation, but it may also be relevant for algorithms that are based on the permutation of starting values, as, for example, gradient projection algorithms (Bernaards and Jennrich, 2005).

From an applied perspective, it should be noted that the correlation of the factor score predictors with the original factors (i.e., indeterminacy) was improved by means of OMT-rotation. Reducing the effects of single cross-loadings on the rotation of the loading pattern may therefore also help to improve decisions that are based on individual factor score predictors. As the original factors are typically unknown in applied settings, it is recommended to compare the OT- and the OMT-rotated loading patterns to check whether single observed variables have a theoretically unjustified effect on factor rotation. Note that cross-loadings with smaller effect on oblique rotation may remain substantial after rotation. However, in the OMT-rotated loading pattern, some cross-loadings might remain large while the mean cross-loadings remain close to zero. This would indicate that a balanced measurement of the latent variable is reached without increasing the factor inter-correlations by reducing the single cross-loading. Moreover, correlation-preserving factor score predictors (e.g., McDonald, 1981) also require optimal estimates of the factor inter-correlations. Of course, also in settings where the factor inter-correlations are relevant for further research, OMT-rotation might be considered.

## Data availability statement

Publicly available datasets were analyzed in this study. This data can be found at: http://openpsychometrics.org/_rawdata/.

## Ethics statement

Ethical approval was not required for the study involving humans in accordance with the local legislation and institutional requirements. Written informed consent to participate in this study was not required from the participants or the participants’ legal guardians/next of kin in accordance with the national legislation and the institutional requirements.

## Author contributions

AB: Conceptualization, Investigation, Methodology, Software, Writing – original draft. NH: Conceptualization, Investigation, Software, Writing – review & editing.

## Footnotes

^1^http://openpsychometrics.org/_rawdata/
