# Supplementary material for: Robust oblique Target-rotation for small samples
Source: Front Psychol. 2023 Nov 27;14:1285212. doi: 10.3389/fpsyg.2023.1285212 (PMC10711062; doi:10.3389/fpsyg.2023.1285212)
Supplement: Supplementary file 1 [file Data_Sheet_1.pdf]

Supplementary Material

Robust oblique Target-rotation for small samples

André Beauducel, Norbert Hilger

1 Supplementary Figures

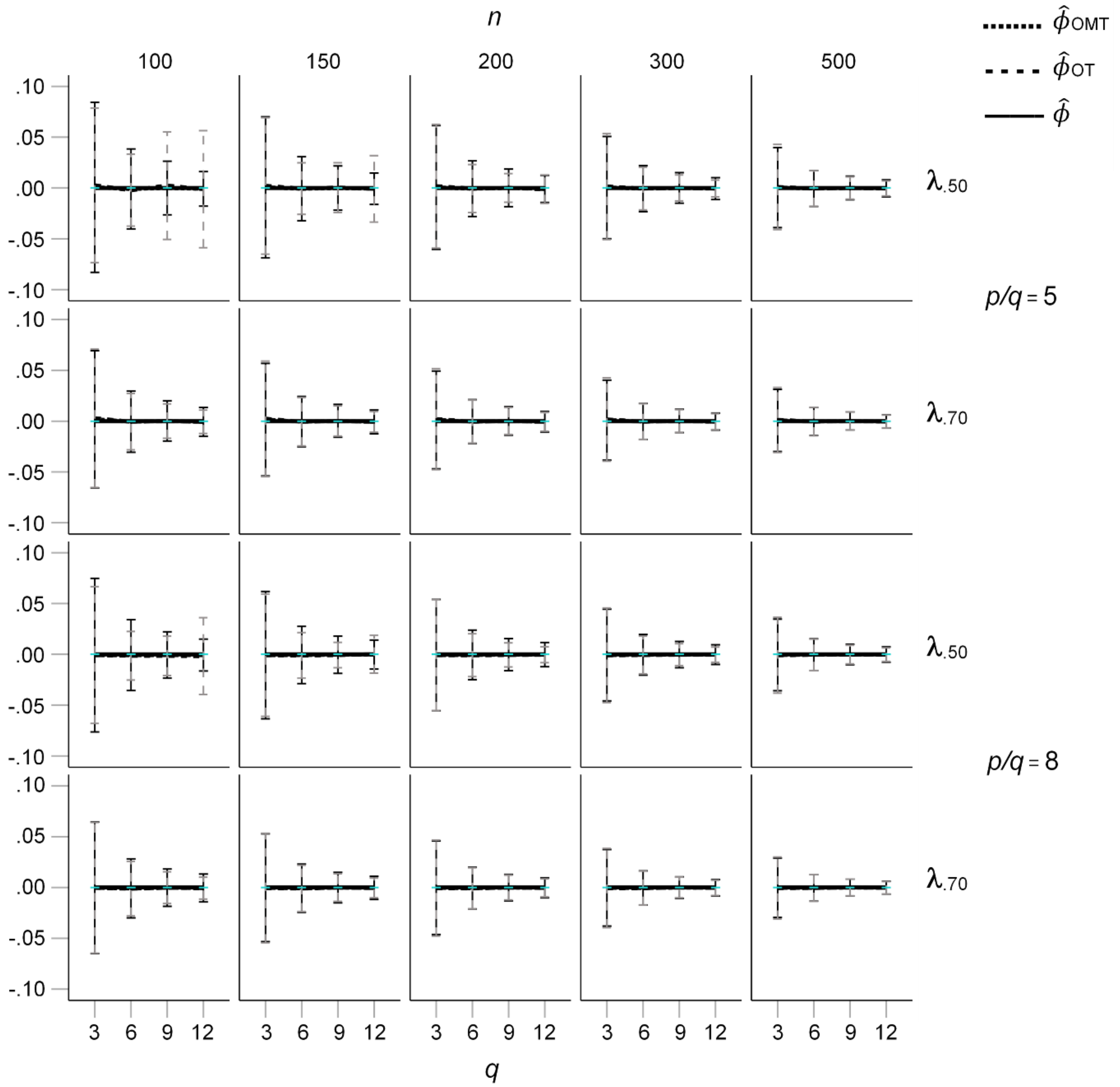

Figure S1. Means and standard deviations of inter-factor correlations resulting from OT- and OMT-rotation for population factor inter-correlations of $\phi = .00$

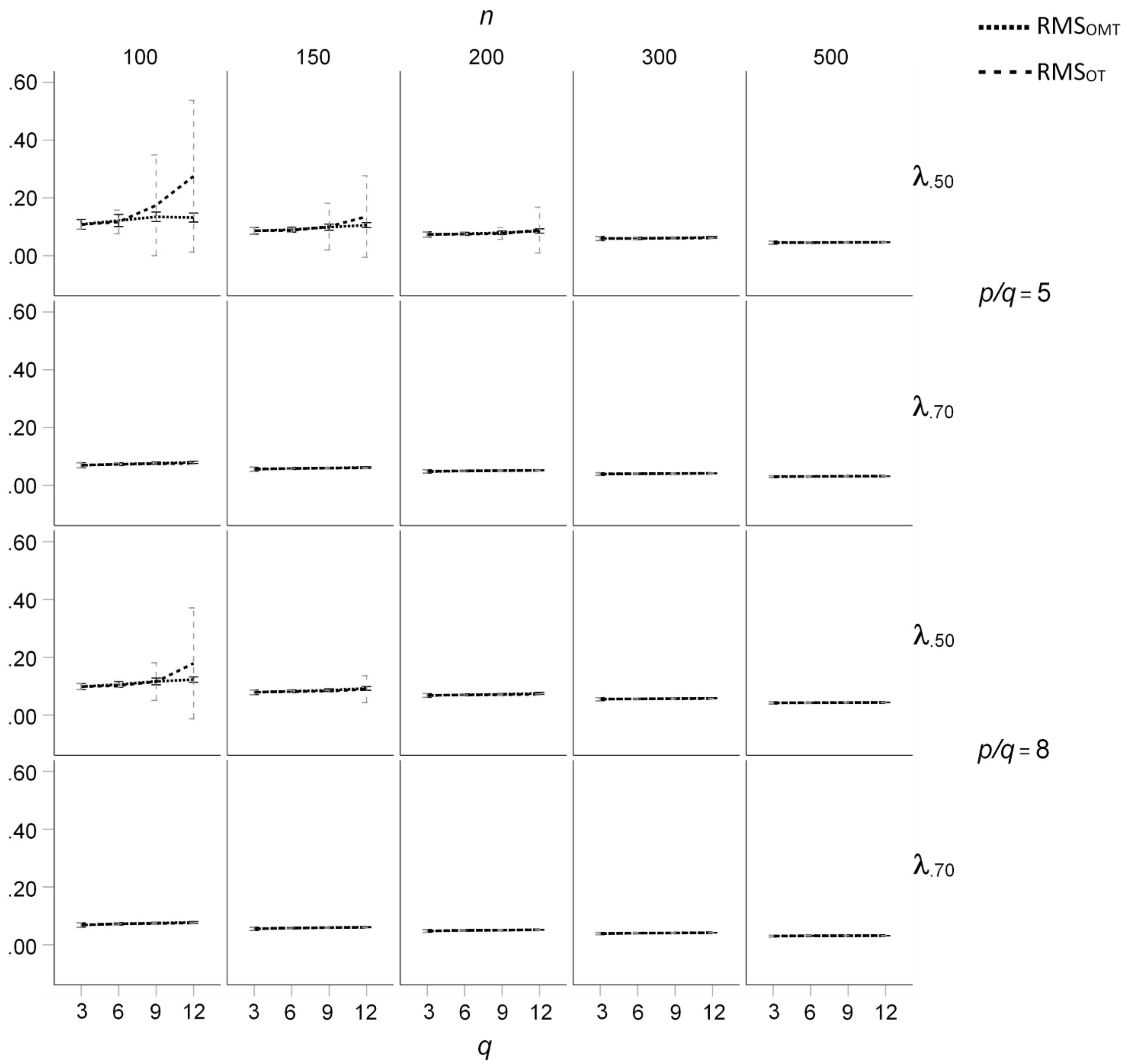

Figure S2. Root Mean Square (RMS) difference between the population loading pattern and the OT- and OMT-rotated loading patterns for population factor inter-correlations of $\phi = .00$

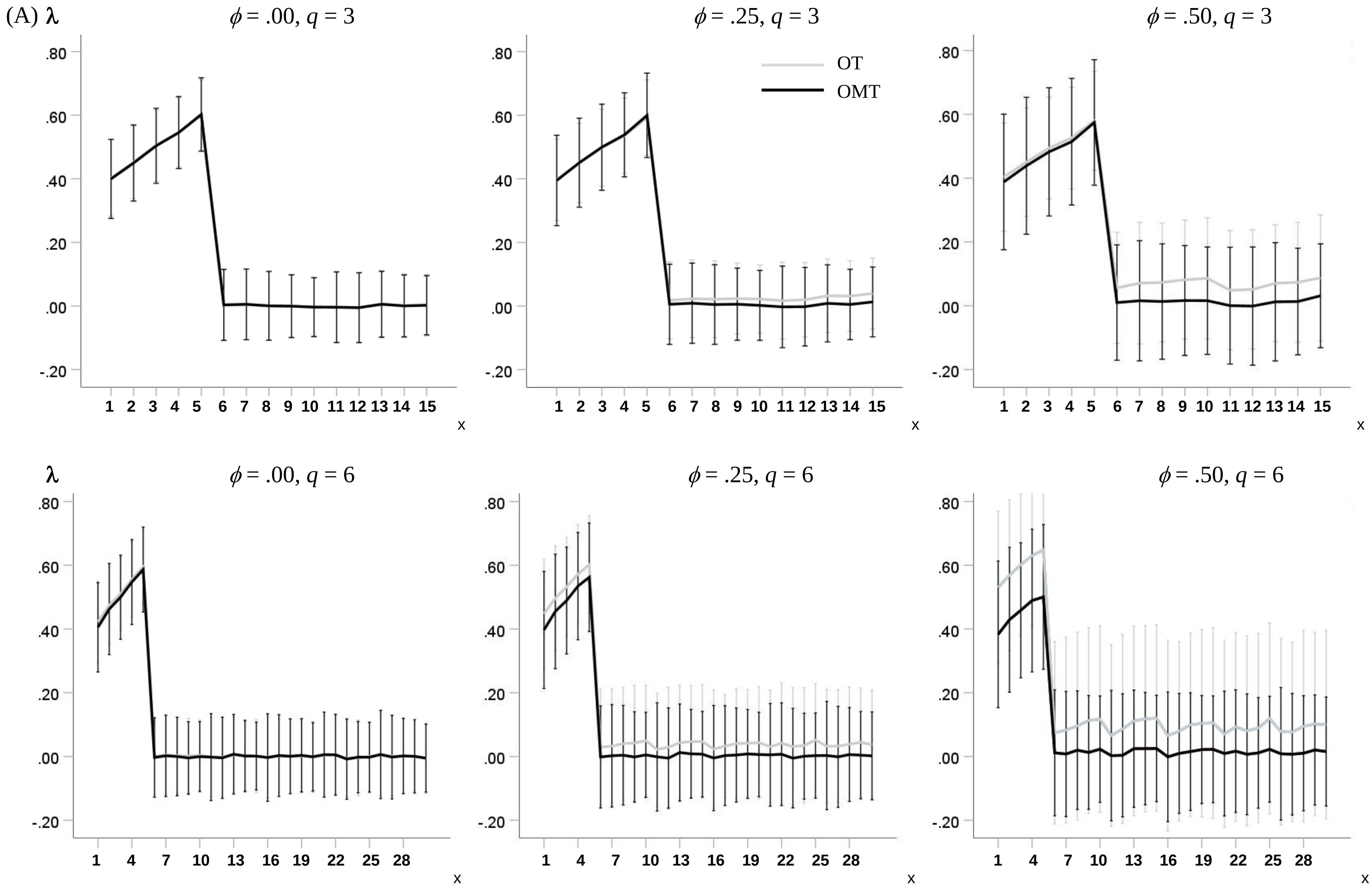

Figure S3 (A). Means and standard deviations of OT- and OMT-rotated loadings of the first factor for ICM based on n = 100, p/q = 5, salient loadings of $\boldsymbol{\lambda}_{.50}$, for q = 3 and q = 6.

(B)

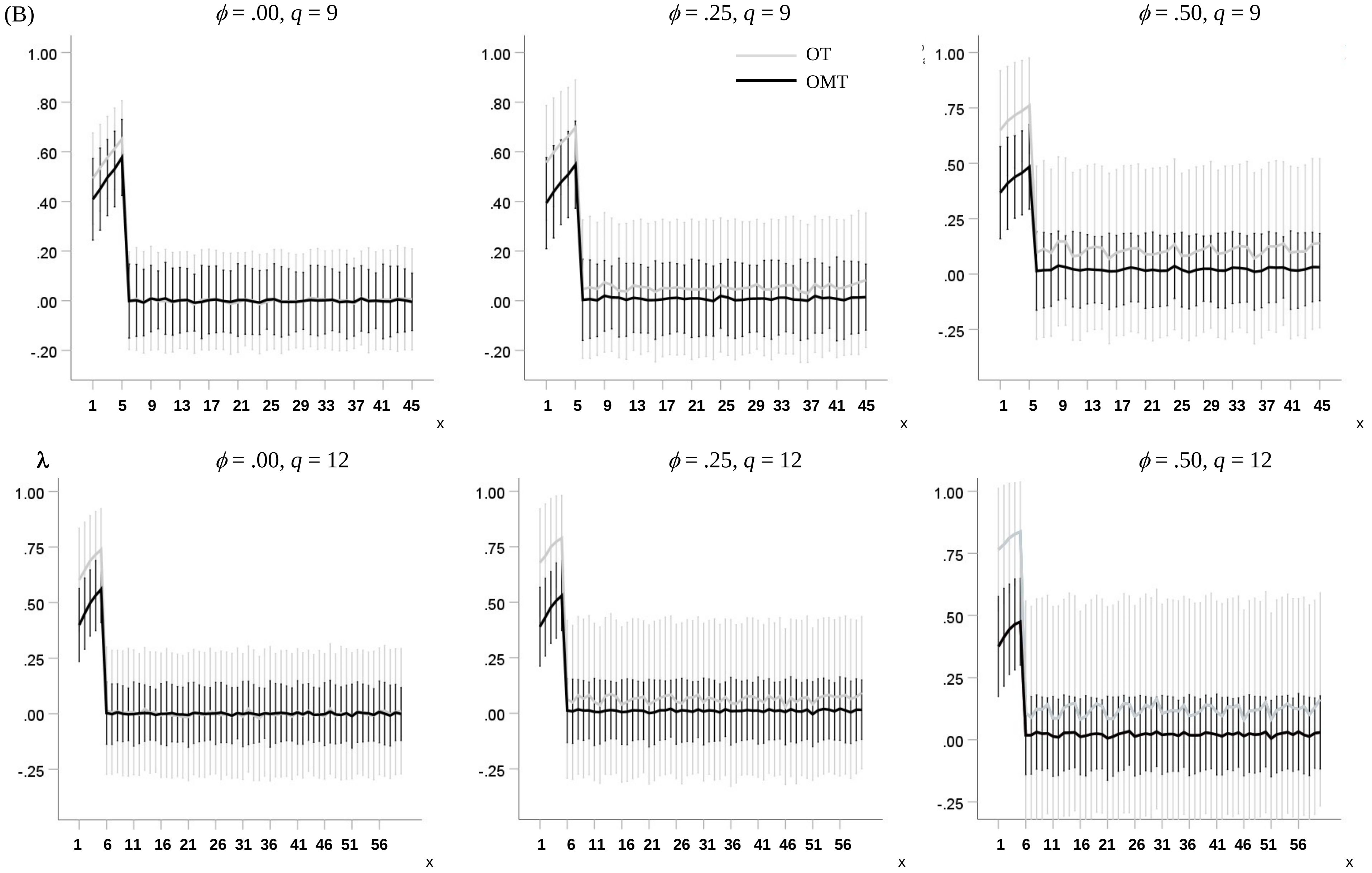

Figure S3 (B). Means and standard deviations of OT- and OMT-rotated loadings of the first factor for ICM based on n = 100, p/q = 5, salient loadings of $\boldsymbol{\lambda}_{.50}$, for q = 9 and q = 12.

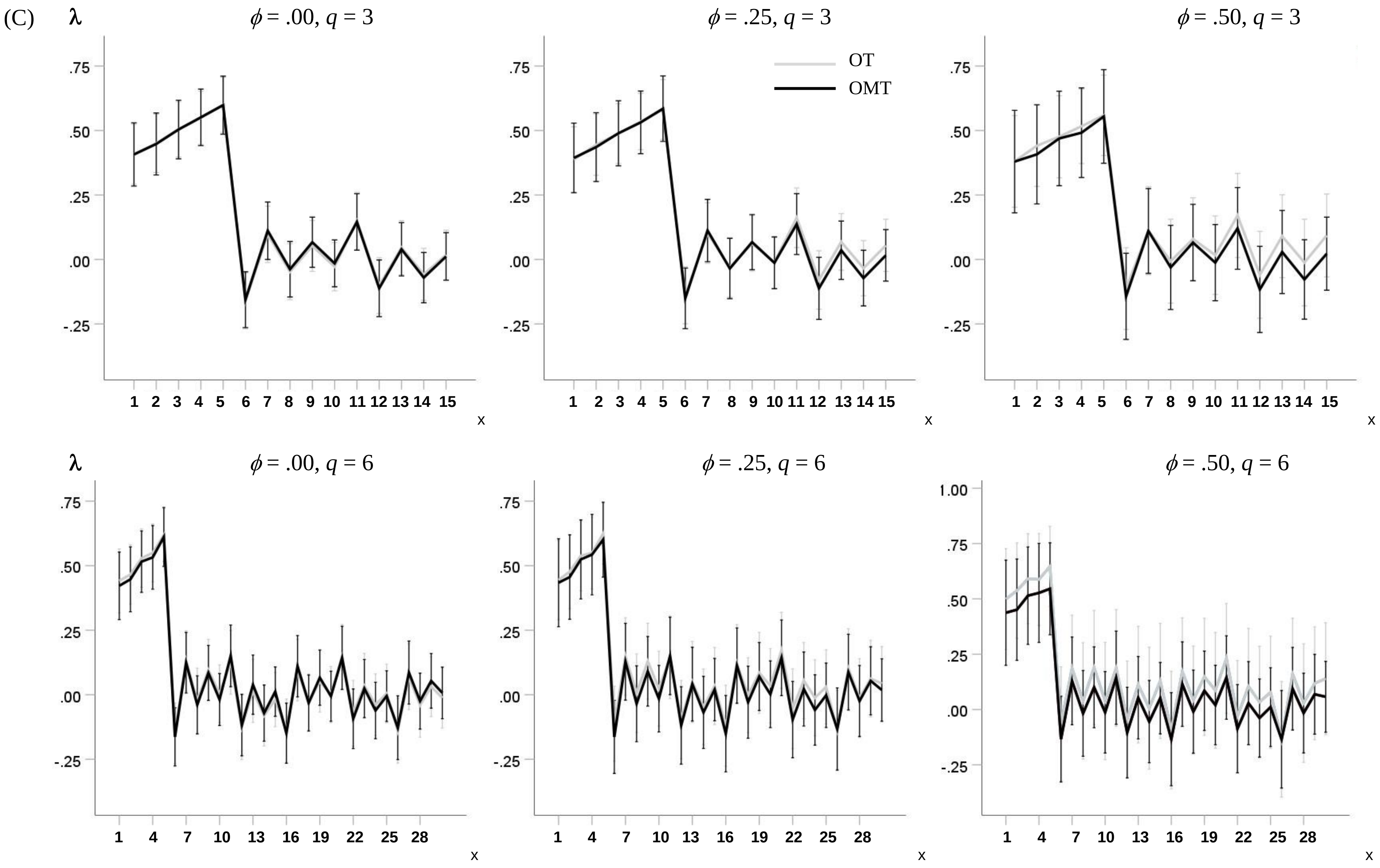

Figure S3 (C). Means and standard deviations of OT- and OMT-rotated loadings of the first factor for ZCLM based on n = 100, p/q = 5, salient loadings of $\boldsymbol{\lambda}_{.50}$, for q = 3 and q = 6.

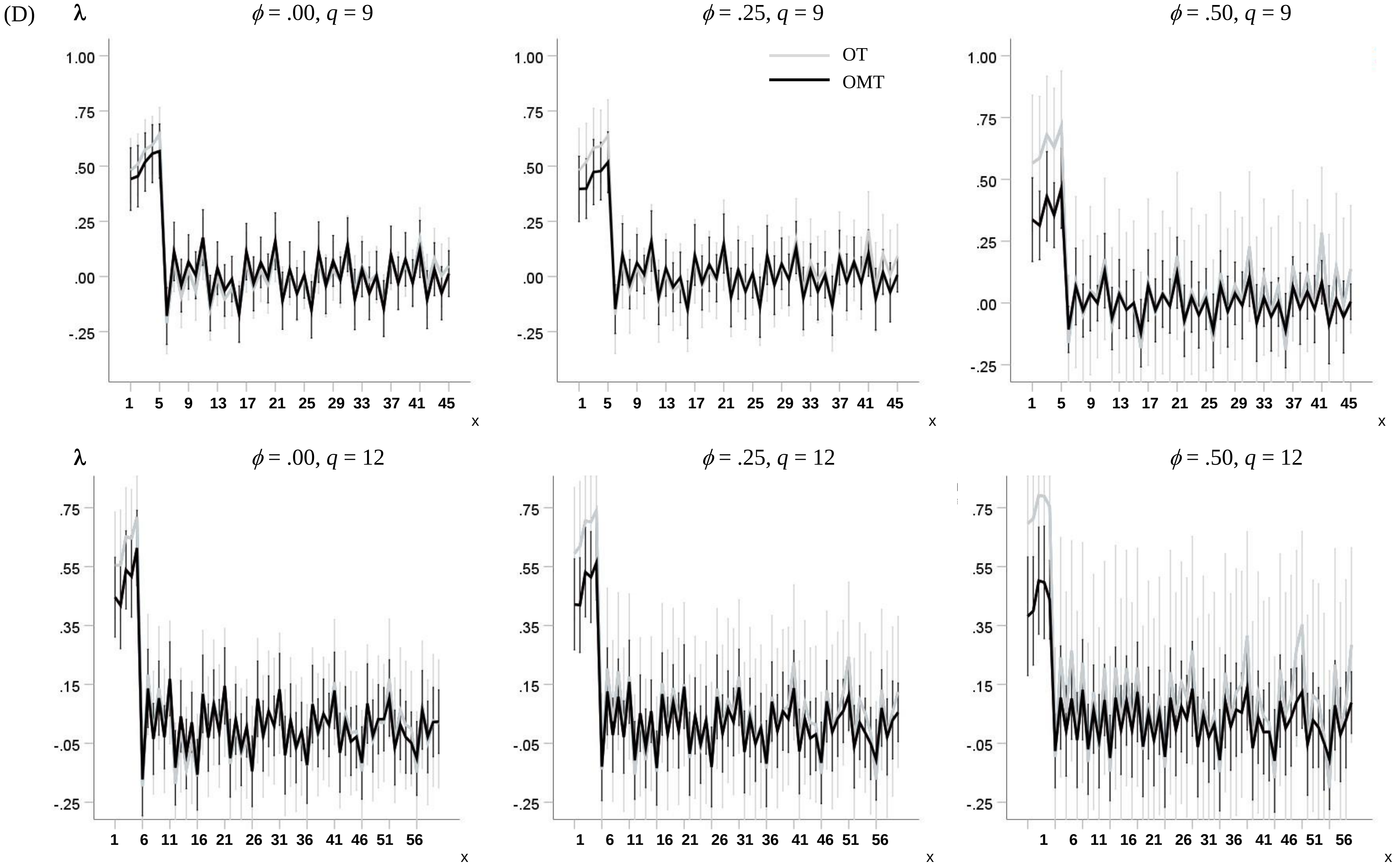

Figure S3 (D). Means and standard deviations of OT- and OMT-rotated loadings of the first factor for ZCLM based on n = 100, p/q = 5, salient loadings of $\boldsymbol{\lambda}_{.50}$, for q = 9 and q = 12.

2 Codes

```
# R-Script:
# Context: R-4.3.1, the following packages are needed:
library(ramify)
library(RSpectra)
library(matrixcalc)
library(fastmatrix)

# Helper functions for frequently used matrix operations
Mdiag <- function(x) return(diag(diag(x)))
inv <- function(x) return(solve(x))
helpPhi <- function(x) return( inv(t(x)%*%x)%*%t(x)%*% L%*%t(L) %*%x%*%inv(t(x)%*%x) )


# Enter values for Oblique Mean Target (OMT)-Rotation:

# number of factors:
q <- 3

# number of variables:
p <- 18

# Enter repetitions for Ridge-constant (reducing Kappa):
Iterate <- 100

# Enter Kappa that might be regarded as too large (default = 20):
k_level <- 20


# Enter inital orthogonal loadings L_u for Target-rotation.

L <- matrix(0, nrow= p, ncol= q)
L[1,]  <- c( 0.50, 0.20,-0.20)
L[2,]  <- c( 0.50,-0.20, 0.20)
L[3,]  <- c( 0.50, 0.20,-0.20)
L[4,]  <- c( 0.50,-0.20, 0.20)
L[5,]  <- c( 0.50, 0.20,-0.20)
L[6,]  <- c( 0.50,-0.20, 0.20)
L[7,]  <- c( 0.20, 0.50,-0.20)
L[8,]  <- c(-0.20, 0.50, 0.20)
L[9,]  <- c( 0.20, 0.50,-0.20)
L[10,] <- c(-0.20, 0.50, 0.20)
L[11,] <- c( 0.20, 0.50,-0.20)
L[12,] <- c(-0.20, 0.50, 0.20)
L[13,] <- c( 0.20,-0.20, 0.50)
L[14,] <- c(-0.20, 0.20, 0.50)
L[15,] <- c( 0.20,-0.20, 0.50)
L[16,] <- c(-0.20, 0.20, 0.50)
L[17,] <- c( 0.20,-0.20, 0.50)
L[18,] <- c(-0.20, 0.20, 0.50)
print(round(L,2))

# Orthogonal factors:
Phi <- diag(1,q)



# Enter ICM-Target-matrix: "1" for salient-loading, "0" for non-salient loadings.

# For block-diagonal Target-matrices you may use:.
```

```
#IDmat <- diag(1,q)
#Tar <- kronecker.prod(IDmat, matrix(1,p/q,1))
# More complex Target-matrices can be entered directly:.
Tar <- matrix(0, nrow= p, ncol= q)
Tar[1,]  <- c(1, 0, 0)
Tar[2,]  <- c(1, 0, 0)
Tar[3,]  <- c(1, 0, 0)
Tar[4,]  <- c(1, 0, 0)
Tar[5,]  <- c(1, 0, 0)
Tar[6,]  <- c(1, 0, 0)
Tar[7,]  <- c(0, 1, 0)
Tar[8,]  <- c(0, 1, 0)
Tar[9,]  <- c(0, 1, 0)
Tar[10,] <- c(0, 1, 0)
Tar[11,] <- c(0, 1, 0)
Tar[12,] <- c(0, 1, 0)
Tar[13,] <- c(0, 0, 1)
Tar[14,] <- c(0, 0, 1)
Tar[15,] <- c(0, 0, 1)
Tar[16,] <- c(0, 0, 1)
Tar[17,] <- c(0, 0, 1)
Tar[18,] <- c(0, 0, 1)
#print(round(Tar,2))

# Oblique Mean Target (OMT)-Rotation:
#1 orthogonal Target-rotation, Schönemann, 1966:

S <- t(L)%*%(Tar)
help1e <- eigen(S%*%t(S))
WW <- help1e$vectors
help2 <- t(S)%*%S
help2e <- eigen(help2)
V <- help2e$vectors
O <- t(WW)%*%S%*%V
ON <- ((O)/abs(O+0.00000000000001))
K <- diag(diag(ON))
WWW <- (WW%*%K)
TR <- WWW%*%t(V)
L1 <- L%*%TR

#2 weigthed average loadings per ICM-cluster according to Equation 4:
L1m <- t(L1 * Tar) %*% (L1) %*% inv(t(L1 * Tar) %*% Tar)
L1m
# Target-matrix for oblique Target-rotation
Iq <- diag(1,q)

#3 oblique rotation of averaged loadings, Target-rotation, Hurley & Cattell, 1962:
# Equation 5:
cong_OMT_old <- 0
help <- t(L1m)%*%L1m
for (ii in 1:Iterate) {
if (kappa(help) > k_level) {
help <- help + diag(0.01,q)
}
T <- inv(help)%*%t(L1m)%*%Iq
# normalize transformation matrix, Equation 6:
Tn <- inv(Mdiag(t(T)%*%T)^0.5)%*%T
# reference structure, Equation 7:
L2 <- L1%*%Tn
# OMT- loading pattern according to Equation 8:
L_OMT <- L2 %*% Mdiag(inv(t(Tn)%*%Tn))^0.5
# Equation 9:
cong_OMT <- matrix.trace( t(L_OMT)%*%Tar  %*%inv( Mdiag(t(L_OMT)%*%L_OMT) %*%
       Mdiag(t(Tar)%*%Tar) )^0.5) / q
```

```
if (cong_OMT > cong_OMT_old) {
L_OMT_old <- L_OMT
cong_OMT_old <- cong_OMT
}
}
cong_OMT <- cong_OMT_old
L_OMT <- L_OMT_old
Phi_OMT <- helpPhi(L_OMT)
round(L_OMT,2)
round(Phi_OMT,2)
round(cong_OMT, 3)



# Oblique Target-rotation (OT), Hurley & Cattell, 1962:
TT <- inv(t(L)%*%L)%*%t(L)%*%Tar
check <- t(TT)%*%TT
D <- Mdiag(check)^0.5
# normalize transformation matrix
TT <- inv(D)%*%TT
# reference structure
FTT <- L%*%TT
CR <- t(TT)%*%TT
D <- inv(Mdiag(inv(CR))^0.5)
# factor pattern and phi
L_OT <- FTT%*%inv(D)
Phi_OT <- helpPhi(L_OT)
round(L_OT,2)
round(Phi_OT,2)
cong_OT <- matrix.trace( t(L_OT)%*%Tar  %*%inv( Mdiag(t(L_OT)%*%L_OT) %*%
       Mdiag(t(Tar)%*%Tar) )^0.5) / q
round(cong_OT, 3)
```

```
* SPSS-Script:

* Encoding: windows-1252.

SET MXLOOPS = 100.
MATRIX.

/* Enter values for Oblique Mean Target (OMT)-Rotation .
/* Enter repetitions for Ridge-constant (reducing kappa) .
compute Iterate = 100.
/* Enter kappa that might be regarded as too large (default = 20) .
compute k_level = 20.
/* Enter inital orthogonal loadings L_u for Target-rotation .
compute L_u = {.50,  .20, -.20;
               .50, -.20,  .20;
               .50,  .20, -.20;
               .50, -.20,  .20;
               .50,  .20, -.20;
               .50, -.20,  .20;
               .20,  .50, -.20;
              -.20,  .50,  .20;
               .20,  .50, -.20;
              -.20,  .50,  .20;
               .20,  .50, -.20;
              -.20,  .50,  .20;
               .20, -.20,  .50;
              -.20,  .20,  .50;
               .20, -.20,  .50;
              -.20,  .20,  .50;
               .20, -.20,  .50;
              -.20,  .20,  .50}.
compute p = nrow(L_u).
compute q = ncol(L_u).
/* Enter ICM-Target-matrix: "1" for salient-loading, "0" for non-salient loadings .
/* For block-diagonal Target-matrices you may use:.
/* compute Tar = kroneker(ident(q),make(p/q,1,1)).
/* More complex Target-matrices can be entered directly:.
compute Tar = {1, 0, 0;
               1, 0, 0;
               1, 0, 0;
               1, 0, 0;
               1, 0, 0;
               1, 0, 0;
               0, 1, 0;
               0, 1, 0;
               0, 1, 0;
               0, 1, 0;
               0, 1, 0;
               0, 1, 0;
               0, 0, 1;
               0, 0, 1;
               0, 0, 1;
               0, 0, 1;
               0, 0, 1;
               0, 0, 1
}.
/* Orthogonal factors .
compute phi = ident(q).

/* Oblique Mean Target (OMT)-Rotation .
/* [1] Orthogonal Target-rotation (Schönemann, 1966) .
compute S = t(L_u)*Tar.
call eigen(S*t(S),WW,lamb).
call eigen(t(S)*S,V,lamb).
```

```
compute O = t(WW)*S*V.
compute ON = ((O)/abs(O+1E-14)).
compute K = mdiag(diag(ON)).
compute WWW = WW*K.
compute TR = WWW*t(V).
compute L1 = L_u*TR.

/* [2] Weigthed average loadings per ICM-cluster (Equation 4) .
compute L1m = t(L1&*Tar)*(L1)*inv(t(L1&*Tar)*Tar).
/* Target-matrix for oblique Target-rotation .
compute Iq = mdiag(make(q,1,1)).

/* [3] Oblique rotation of averaged loadings, Target-rotation (Hurley & Cattell, 1962) .
/* Equation 5 .
compute cong_OMT_old = 0.
compute help = t(L1m)*L1m.
loop ii = 1 to Iterate.
   compute T_ = inv(help)*t(L1m)*Iq.
/* Normalize transformation matrix (Equation 6) .
   compute Tn = inv(mdiag(diag(t(T_)*T_))&**0.5)*T_.
/* Reference structure (Equation 7) .
   compute L2 = L1*Tn.
/* OMT-loading pattern (Equation 8) .
   compute L_OMT = L2*mdiag(diag(inv(t(Tn)*Tn)))&**0.5.
/* Equation 9 .
   compute cong_OMT =
       trace(t(L_OMT)*Tar&*inv(mdiag(diag(t(L_OMT)*L_OMT))&*mdiag(diag(t(Tar)*Tar)))&**0.5)/
       q.
   call eigen(help,vec,val).
   compute kappa = abs(mmax(val))/abs(mmin(val)).
   do if (cong_OMT > cong_OMT_old).
      compute L_OMT_old = L_OMT.
      compute cong_OMT_old = cong_OMT.
   end if.
   do if kappa > k_level.
      compute help = help+mdiag(make(q,1,0.01)).
   else.
      break.
   end if.
end loop.
compute cong_OMT = cong_OMT_old.
compute L_OMT = L_OMT_old.
compute Phi_OMT = inv(t(L_OMT)*L_OMT)*t(L_OMT)*L_u*t(L_u)*L_OMT*inv(t(L_OMT)*L_OMT).

/* Oblique Target-rotation (OT) (Hurley & Cattell, 1962) .
compute TT = inv(t(L_u)*L_u)*t(L_u)*Tar.
compute check = t(TT)*TT.
compute D = mdiag(diag(check))&**0.5.

/* Normalize transformation matrix .
compute TT = inv(D)*TT.

/* Reference structure .
compute FTT = L_u*TT.
compute CR = t(TT)*TT.
compute D = inv(mdiag(diag(inv(CR)))&**0.5).

/* Factor pattern and phi .
compute L_OT = FTT*inv(D).
compute Phi_OT = inv(t(L_OT)*L_OT)*t(L_OT)*L_u*t(L_u)*L_OT*inv(t(L_OT)*L_OT).
compute cong_OT =
       trace(t(L_OT)*Tar&*inv(mdiag(diag(t(L_OT)*L_OT))&*mdiag(diag(t(Tar)*Tar)))&**0.5)/q.

print L_u /formats=f5.2.
```

```
print L1m /formats=f5.2.
print L_OMT /formats=f5.2.
print Phi_OMT /formats=f5.2.
print cong_OMT /formats=f5.2.
print L_OT /formats=f5.2.
print Phi_OT /formats=f5.2.
print cong_OT /formats=f5.2.

END MATRIX.
```
